# Electrochemical Properties of Powdery LiNi_1/3_Mn_1/3_Co_1/3_O_2_ Electrodes with Styrene-Acrylic-Rubber-Based Latex Binders at High Voltage

**DOI:** 10.1021/acsami.4c11185

**Published:** 2024-11-25

**Authors:** Lu Yin, Ryoichi Tatara, Kosuke Nakamoto, Shogo Yamazaki, Rena Takaishi, Eisuke Shiiyama, Takashi Matsuyama, Shinichi Komaba

**Affiliations:** † Department of Applied Chemistry, Tokyo University of Science, 1-3 Kagurazaka, Shinjuku, Tokyo 162-8601, Japan; ‡ NIPPON A&L INC, 3-1-98 Kasugadenaka, Konohanaku, Osaka 554-8558, Japan

**Keywords:** lithium-ion batteries, latex binders, acrylic rubbers, layered oxides, LiNi_1/3_Mn_1/3_Co_1/3_O_2_, LiNi_0.6_Mn_0.2_Co_0.2_O_2_

## Abstract

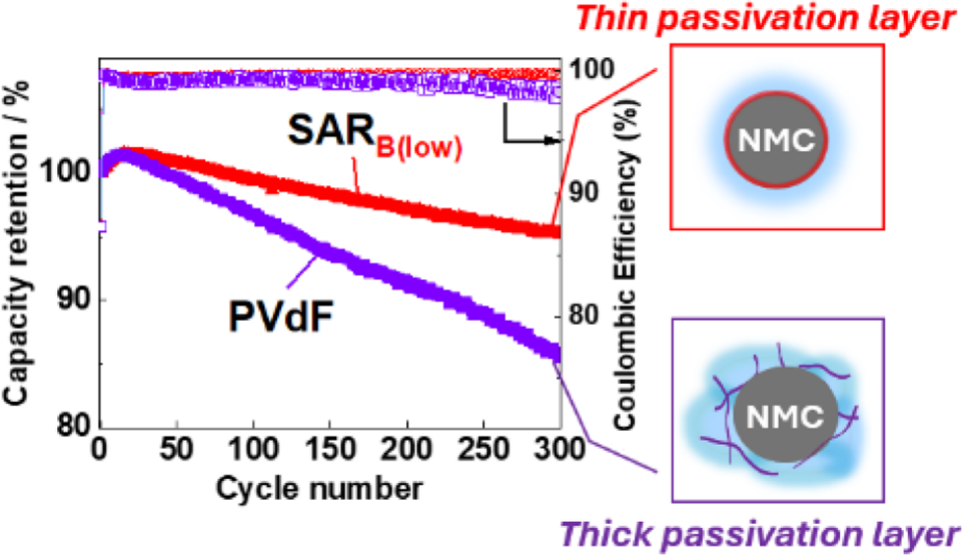

Efforts to improve the energy density and cycling stability of lithium-ion batteries have focused on replacing LiCoO_2_ in cathodes with LiNi_*x*_Mn_*y*_Co_1–*x*–_*_y_*O_2_. However, reliance on polyvinylidene fluoride (PVdF) as the binder limits the application of the LiNi_*x*_Mn_*y*_Co_1–*x*–_*_y_*O_2_ composite electrode for lithium-ion batteries. Here, we evaluate the electrochemical properties of a LiNi_1/3_Mn_1/3_Co_1/3_O_2_ (NMC111) powder electrode formed using a waterborne-styrene-acrylic-rubber (SAR) latex binder combined with sodium carboxymethylcellulose. The composite electrodes prepared with the SAR-based binder copolymerized with the butyl acrylate monomer and styrene exhibited high adhesive strength and excellent cyclability and rate capability. The results of surface analysis via X-ray photoelectron spectroscopy suggested that the electrode with the SAR-based binder is more resistant to electrolyte decomposition during charge and discharge cycling compared with the NMC111 electrode comprising the conventional PVdF binder. The SAR-derived passivation resulted in enhanced capacity retention during long-term cycling tests of both half- and full-cells (NMC111//graphite). An electrode with a higher Ni content, LiNi_0.6_Mn_0.2_Co_0.2_O_2_ (NMC622), fabricated using the SAR-based binder, retained 87.1% of its capacity after 50 cycles at 4.6 V and exhibited excellent cycling stability.

## Introduction

The demand for lithium-ion batteries (LIBs) with high energy density and excellent cycling stability is being driven by the growing commercialization of electric vehicles.^1^ In an attempt to improve the performance of these batteries, researchers have recently turned their attention to layered oxide LiNi_1/3_Mn_1/3_Co_1/3_O_2_ (NMC111) as the positive electrode material for LIBs owing to its higher capacity and lower cost than LiCoO_2_ cathodes.^2−6^ However, the higher initial irreversible capacity of NMC111 than that of LiCoO_2_ rapidly increases when charged at a voltage of >4.3 V.^7^ Other disadvantages associated with this electrode are (i) the appearance of microcracks during charge–discharge cycling, (ii) leaching of transition metals, and (iii) irreversible transitions to the rock-salt phase; thus, they adversely affect the cycling performance of the cells with NMC. Because of its higher Ni content, NMC tends to react with water; thus, hydrophobic polyvinylidene fluoride (PVdF) has been widely used as a binder.^8−14^ However, PVdF must be dissolved in *N*-methyl-2-pyrrolidone (NMP), a volatile and toxic organic solvent that is an environmental pollutant.^15^ Efforts to circumvent this problem include the addition of acid during slurry preparation to enable the use of an aqueous binder,^16−19^ the application of a coating to the surface of active material particles,^20,21^ and the replacement of an aluminum current collector with carbon fabric.^22^

Previous studies aiming to replace PVdF have explored the use of water-based binders, styrene butadiene rubber (SBR) and sodium carboxymethylcellulose (CMC), in graphite anodes of practical LIBs. Previously, we demonstrated that the employment of SBR/CMC binders in LiCoO_2_ electrodes through water processing resulted in enhanced cycling performance compared to that of PVdF-based electrode.^23^ SBR-based binders with different cross-link densities and functional groups have also been prepared.^24−26^ Despite their advantages, a notable drawback of SBR-based binders is their lack of oxidative stability due to the oxidation of the unsaturated C=C double bonds of the butadiene group in SBR. To overcome this problem, we developed a new type of acrylic rubber binder that does not contain any C=C double bonds.^27^ In this study, we prepared a series of styrene acrylic rubber (SAR) latex via the polymerization of butyl acrylate (SAR_B_) and 2-ethylhexyl acrylate (SAR_2EH_) with styrene to examine them as binders and focus on the difference in their molecular structures and cross-link densities.^28^ Furthermore, we evaluated the impact of these different SAR-based binders on the electrochemical performance of LiNi_*x*_Mn_*y*_Co_1–*x*–*y*_O_2_ electrodes in a high-voltage setting.

## Experimental Section

### Materials

**Figure 1:**
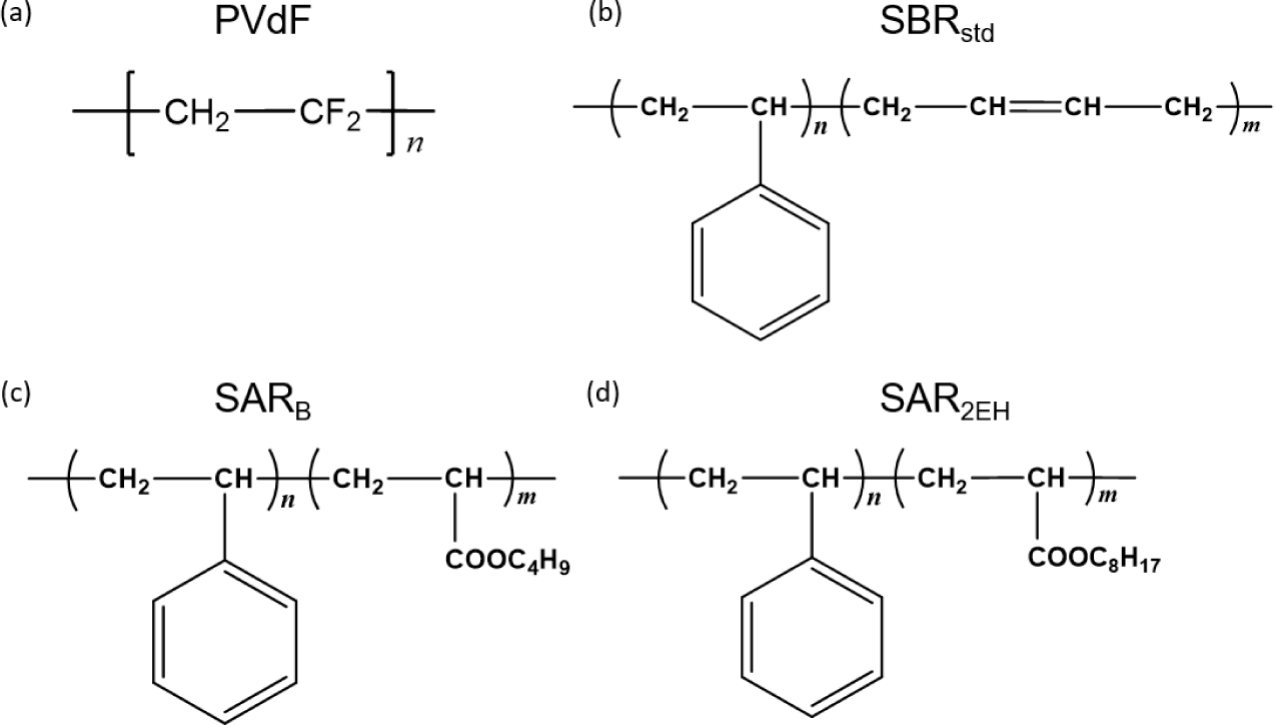
Molecular structures of the binder materials: (a) poly(vinylidene fluoride) (PVdF), (b) styrene butadiene rubber (SBR_std_), (c) styrene-acrylic-rubber (copolymer to which the butyl acrylate monomer was introduced) (SAR_B_), and (d) styrene-acrylic-rubber (copolymer to which the 2-ethylhexyl acrylate monomer was introduced) (SAR_2EH_).

Aqueous dispersions of all SAR-based latex binders were developed by NIPPON A&L Inc. Four different types of binders, labeled SAR_B(high)_, SAR_B(low)_, SAR_2EH(high)_, and SAR_2EH(low)_, where “low” and “high” denote low and high degrees of cross-linking, respectively. Samples SAR_B_ and SAR_2EH_ were synthesized through copolymerizing a butyl acrylate and 2-ethylhexyl acrylate monomer with styrene, respectively, and by changing the degree of cross-linking (Figure 1).^28^ NMC111 was supplied by Nippon Chemical Industrial Co., Ltd. LiNi_0.6_Mn_0.2_Co_0.2_O_2_ (Tokyo Chemical Industry Co., Ltd.), acetylene black (AB; Denka Black Li-400, Denka Co. Ltd.), CMC (CMC#2200, Daicel Miraizu Ltd.), and PVdF (Polysciences Co.) were used in their original form. NMP (purity >99.0%, Kanto Chemical Co., Ltd.) and a solution of LiPF_6_ (1.0 mol dm^–3^) in ethylene carbonate (EC) and dimethyl carbonate (DMC) (volume ratio, EC/DMC = 1/1, lithium battery grade, Kishida Chemical Co., Ltd.) were used as received. Deionized water (DI) with a conductivity of <1.0 μS cm^–1^ was obtained using a purification system (Purelite, PRA-0015, Organo).

### Preparation of Electrodes

NMC-based composite electrodes were prepared by thoroughly blending [LiNi_1/3_Mn_1/3_Co_1/3_O_2_ or LiNi_0.6_Mn_0.2_Co_0.2_O_2_], AB, [SAR_*x*_ or SBR_std_], and CMC thickener in a mass ratio of 80:10:0.5:0.5 (corresponding to 87.9:11.0:0.55:0.55 wt %) in DI water. Conventional LiCoO_2_ powder electrodes were also prepared by thoroughly blending LiCoO_2_, AB, SAR_*x*_, and CMC in a mass ratio of 80:10:0.5:0.5 with DI water, and by blending a mixture of LiCoO_2_, AB, SBR_std_, and CMC in a mass ratio of 80:10:0.5:1.5 (corresponding to 87.0:10.9:0.5:1.6 wt %) with adequate amount of DI water. Negative electrodes were fabricated by thoroughly mixing graphite, SBR_std_, and CMC in a mass ratio of 90:1:3 in DI water, and by mixing the active materials, AB, and PVdF in a mass ratio of 80:10:10 by adding an adequate amount of NMP as the solvent. It should be noted that a larger quantity of binder was required for the PVdF-based electrode to achieve sufficient mechanical strength than that for the SAR and SBR-based electrodes. The resulting homogeneous slurries were coated onto aluminum foil current collectors using a doctor blade and dried under vacuum at 80 °C. The mass loading of LiNi_*x*_Mn_*y*_Co_1–*x*–_*_y_*O_2_ ranged between 3.6 and 3.9 mg cm^–2^.

### Characterization

Galvanostatic charge–discharge tests were performed using R2032-type coin cells assembled in an Ar-filled glovebox (Miwa). Battery grade Li metal foil (Honjo) was used as the counter electrode in half-cell tests (NMC//Li), whereas full-cell tests were conducted using graphite as the negative electrode (NMC//graphite) with a 5–15% excess capacity loaded on the graphite side. A porous polyolefin sheet was used as the separator. Half- and full-cells were tested in the voltage ranges of 3.0–4.6 and 3.0–4.5 V, respectively. The cells were cycled at a current rate of C/10 (27.41 mA g^–1^) at room temperature of about 25 °C. Cyclic voltammetry of the NMC-free AB/binder electrodes was performed at a scan rate of 0.25 mV s^–1^ in the voltage range of 3.0–5.0 V in order to evaluate anodic stability of binder by preparing electrodes consisting of AB:PVdF = 80:20, AB:SBR_std_:CMC = 80:10:10, and AB:SAR_*x*_:CMC = 80:10:10 (m/m) formed on an aluminum current collector. The solvent uptake and solubility of the polymer binders were determined by measuring the change in the mass after immersing the pure polymer films (thickness: 3 mm) in an EC/DMC solvent mixture. Binder films (width: 1.0 cm; length: 1.5 cm; thickness: 50 μm) for the tensile test were prepared by mixing the SAR-based binder and CMC in a ratio of 95:5 (m/m). In the 90° peel test, the adhesive strength of the LiNi_1/3_Mn_1/3_Co_1/3_O_2_ composite electrode was measured using double-sided tape (Scotch Tape, SPS-12, 3 M Japan).^29^ Scanning electron microscopy (SEM, JCM-6000, JEOL, Ltd., Japan) and energy dispersive X-ray spectrometry (EDS; JED-2300) with an acceleration voltage of 15 kV were used to examine the surface morphology of the electrodes. Cross-sectional images were acquired using a cross-section polisher (IB19520CCP, JEOL) for cross-sectional processing and a JEOL JSM-7001F instrument for SEM. X-ray photoelectron spectroscopy (XPS) was performed using a JPS-9010MC (JEOL Ltd.) equipped with a nonmonochromatic Mg Kα X-ray source (1253.6 eV). Electrodes that underwent electrochemical testing were carefully extracted from the cycled coin cells, rinsed with DMC, and dried at room temperature in an argon-filled glovebox. The samples were sealed in an aluminum laminate package inside the glovebox for transport, before they were opened and quickly placed in the chamber to minimize their exposure to air. The binding energies were calibrated using the C 1s peak of the sp^2^ carbon of AB (284.6 eV) as a reference, and the integrated peak intensities were normalized to the same peak after baseline correction.

## Results and Discussion

### Fundamental Properties of SAR-Based Binders

**Figure 2:**
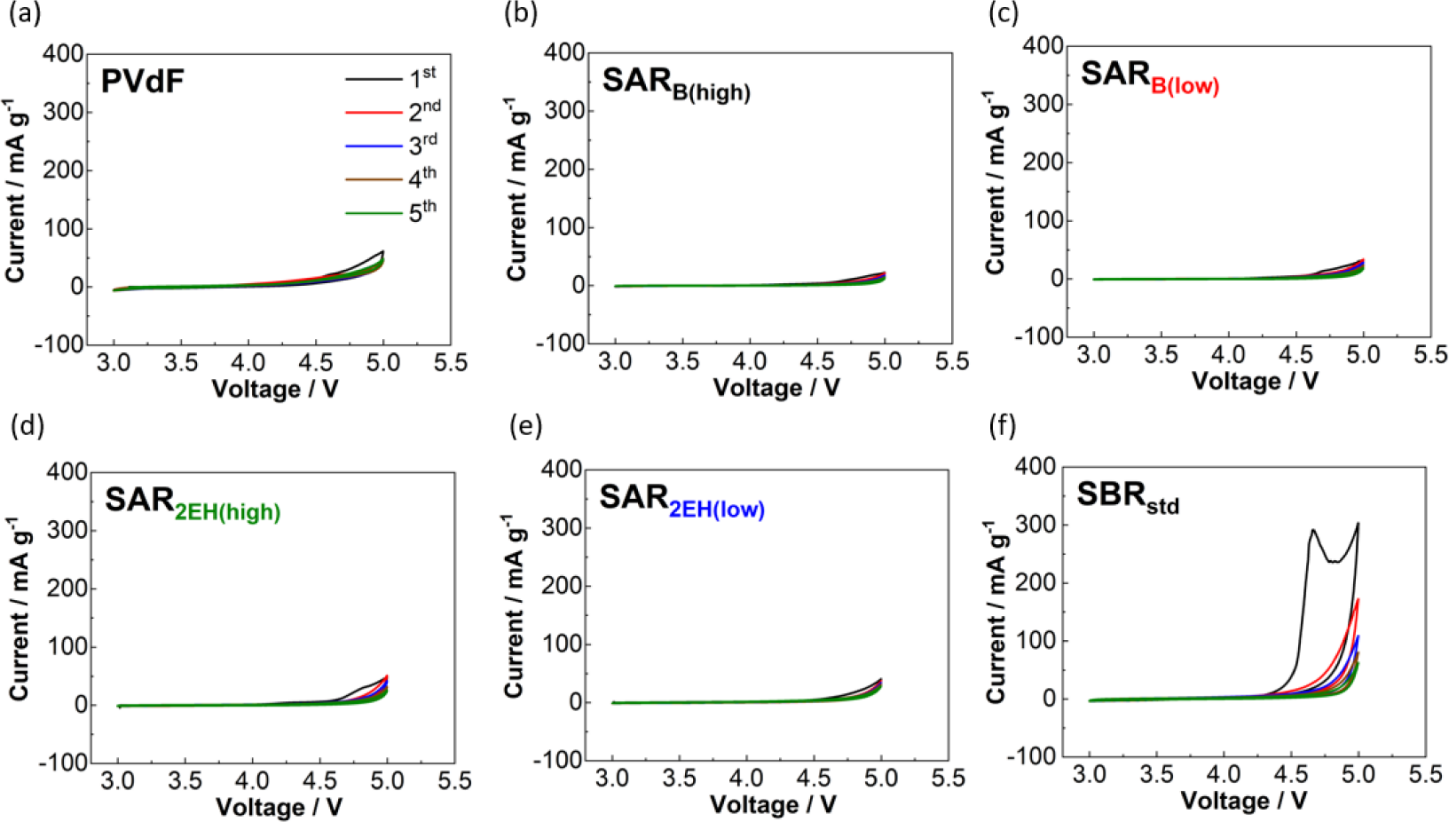
Cyclic voltammograms of the LiNi_1/3_Mn_1/3_Co_1/3_O_2_-free composite electrode containing 80% AB and 20% binder: (a) PVdF, (b) SAR_B(high)_, (c) SAR_B(low)_, (d) SAR_2EH(high)_, (e) SAR_2EH(low)_, and (f) SBR_std_ with 1 mol dm^–3^ LiPF_6_ in EC/DMC as the electrolyte in the voltage range of 3.0–5.0 V at 25 °C.

The C=C bonds in the butadiene moiety constituting SBR_std_ are known to undergo oxidation during the charging process, which lowers the initial Coulombic efficiency.^25,27^ This electrochemical oxidation of the rubber was quantified by subjecting a composite electrode consisting of AB and the binder (i.e., without the active material) to cyclic voltammetry. The electrodes that were prepared with PVdF and the SAR-based binders did not show any apparent oxidation peaks in the cyclic voltammograms (Figure 2), neither did those that incorporated CMC as the binder (Figure S1), confirming that these binders exhibit excellent oxidative stability. In contrast, a remarkable oxidation current of 300 mA g^–1^ was observed in the initial cycles when SBR_std_ was used as the binder; the second and subsequent cycles also gave rise to a higher oxidation current than those achieved when other SAR-based binders were used. This suggested that the initial Coulombic efficiency of the NMC composite with the four SAR-based binders could be expected to be higher than that with the SBR_std_ binder.

The fundamental properties of the binders developed in this study are listed in Table 1. The solvent uptake and solubility of the films of these binders were assessed by immersing the films in an EC/DMC solution.^24^ The SAR_B(low)_ absorbed the most solvent,^28^ and none of the SARs were found to be soluble in this solution. In addition, tensile tests (elongation at break) of the binder films revealed that the tensile stress of the PVdF film was the highest but that deformation became irreversible at low elongation. The SAR_B(low)_ and SAR_2EH(low)_ binders with lower degrees of cross-linking showed higher elasticity and flexibility than those with higher degrees of cross-linking.^28^ Thus, the adhesive strength between the aluminum current collector and composite NMC111 electrodes with SAR as the binder was investigated by conducting a 90° peel test with adhesive tape (Figure S2 and Table 1). The adhesive strength of the SAR-based binders was superior to that of PVdF, which showed a low average load of 0.05 N cm^–1^. In particular, SAR_B_ had the highest load, indicating a significant improvement in mechanical strength. In summary, the excellent fundamental properties of the SAR-based binders were expected to positively affect the cycling stability.

**Table 1:** Physical and Mechanical Properties of Binders Used in This Study

	SAR_B(high)_	SAR_B(low)_	SAR_2EH(high)_	SAR_2EH(low)_	SBR_std_	PVdF
solvent uptake (%)	39^a^	56^a^	42^a^	40^a^	21	20^b^
solubility (%)	4^a^	3^a^	3^a^	3^a^	1	3^b^
elongation at break (mm)	2.2^a^	33.1^a^	9.2^a^	31.3^a^	33.1	2.2^a^
average load (N cm^–1^)	0.49	0.54	0.25	0.31	0.32	0.05

a The data were obtained from ref. (28).

b The data were obtained from ref. (25).

### Electrochemical Performance of NMC111 Electrodes with SAR-Based Binders

The upper cutoff voltage was set to 4.6 V to enable the differences between the different binders to be clearly distinguished. As shown in Figure S3, the capacity retention was higher at lower operating voltages^30,31^ and the cycling performance was independent of the CMC content of the composite electrodes (Figure S4). In this study, electrochemical performance of SAR and SBR-based NMC111 electrodes with 0.5 wt % CMC was evaluated.

**Figure 3:**
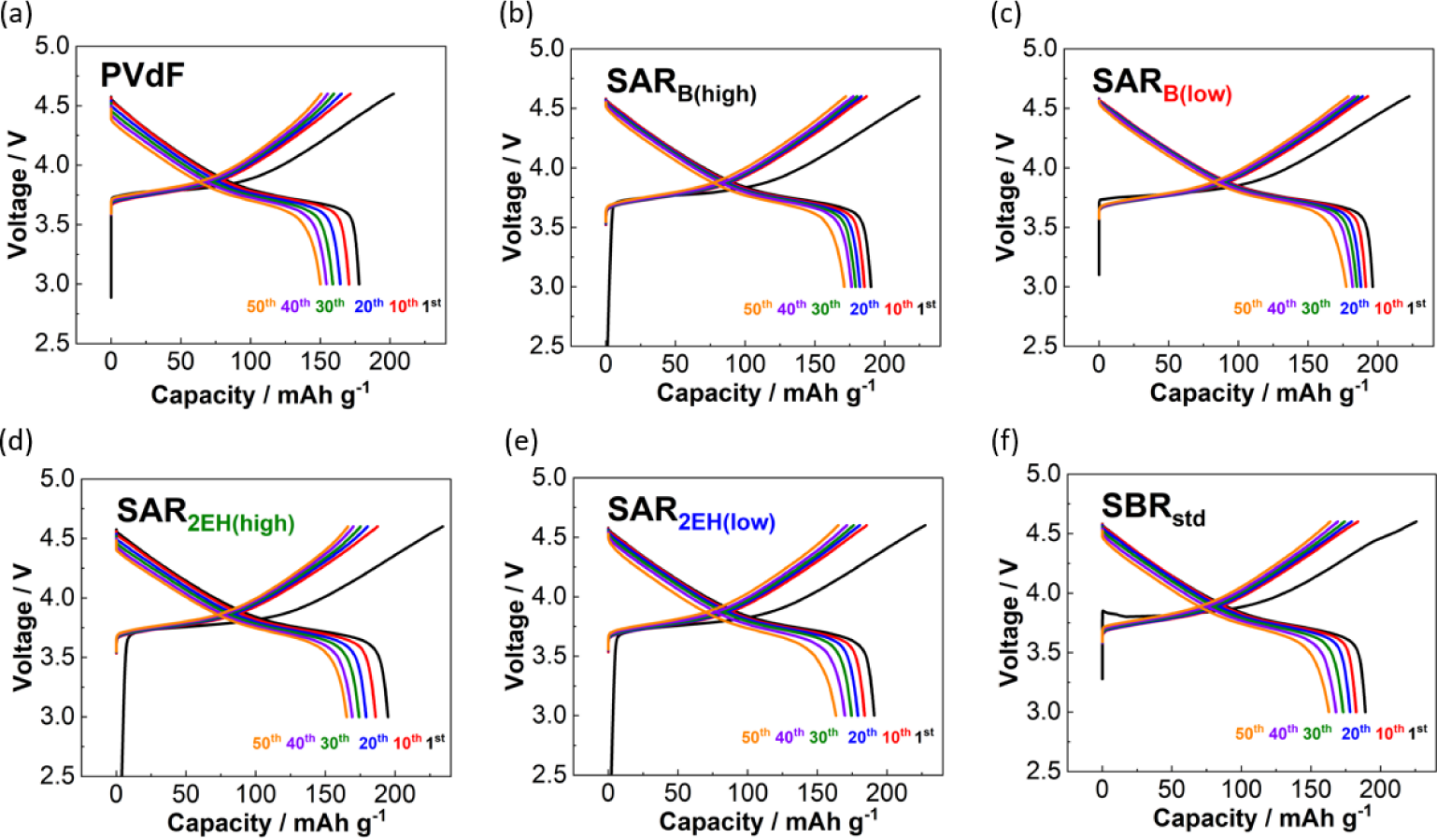
Charge–discharge curves of the LiNi_1/3_Mn_1/3_Co_1/3_O_2_ with (a) PVdF, (b) SAR_B(high)_, (c) SAR_B(low)_, (d) SAR_2EH(high)_, (e) SAR_2EH(low)_, and (f) SBR_std_ as the binders in Li half-cells. The cells were cycled at 27.41 mA g^–1^ at 25 °C and filled with 1 mol dm^–3^ LiPF_6_ in EC/DMC as the electrolyte.

The charge–discharge curves for the NMC111//Li half-cells with the different binders are shown in Figure 3. The discharge capacity of the electrodes prepared with the PVdF, SBR_std_, and SAR_2EH_ binders decreased significantly upon cycling. A high irreversible capacity was observed for the SBR_std_-based electrode (charge and discharge capacities of 226 and 189 mA h g^–1^, respectively, at the first charge–discharge cycle). This irreversibility should be attributed to the oxidation of the C=C double bond in the butadiene moiety of SBR_std_ during the initial charging process.^23,25^

**Figure 4:**
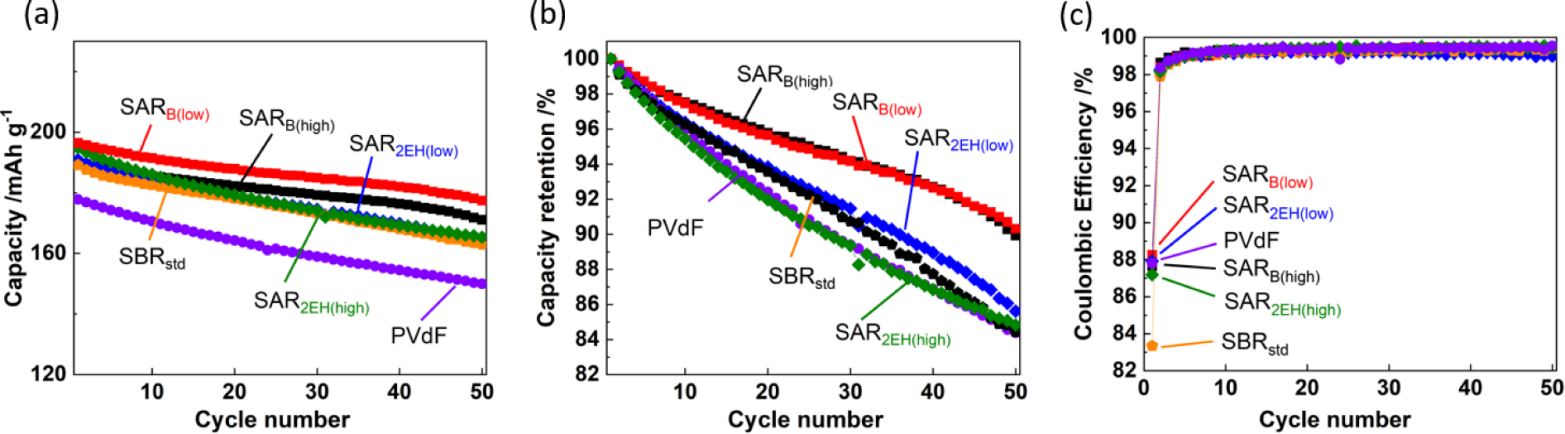
Variation in (a) discharge capacities, (b) capacity retention, and (c) Coulombic efficiencies of the LiNi_1/3_Mn_1/3_Co_1/3_O_2_//Li half-cells cycled at 25 °C with the different binders used in NMC.

The cycling performance of the half-cells with the NMC111 electrodes fabricated with the different binders is illustrated in Figure 4. The electrodes containing SBR_std_ and SAR exhibited higher discharge capacities in the initial cycle than those with PVdF. There are two main reasons for this: (i) previous report has shown that particles (active material, conductive material) are uniformly dispersed without agglomeration in latex binder-based electrodes compared to PVdF-based one,^27^ (ii) the SBR_std_ and SAR-based latex binders, which comprise spherical particles,^32,33^ formed a stronger three-dimensional conductive network than PVdF.^34^ After 50 cycles, the capacity retention of the electrodes containing SAR_B(low)_, SAR_B(high)_, SAR_2EH(low)_, SAR_2EH(high)_, SBR_std_, and PVdF was 90.3%, 89.9%, 85.6%, 84.8%, 84.4%, and 84.4%, respectively. The superior capacity retention of the electrodes fabricated with the SAR-based binders (than that of the PVdF-based electrode) was attributed to their higher adhesive strength. Among them, SAR_B(low)_ achieved higher capacity retention (>90%) even after cycling at 4.6 V for 50 cycles. The initial Coulombic efficiencies decreased in the order SAR_B(low)_, SAR_2EH(low)_, PVdF, SAR_B(high)_, SAR_2EH(high)_, and SBR_std_ (88.3%, 87.9%, 87.8%, 87.6%, 87.2%, and 83.3%, respectively). The low initial Coulombic efficiency of the SBR_std_ electrode was ascribed to the irreversible oxidation of the C=C bond of butadiene at potentials above 4.2 V vs Li^+^/Li, as shown in Figure 2.^24−28^ Nonetheless, the Coulombic efficiency of the SBR_std_ electrode increased from the second cycle onward, with average Coulombic efficiencies of >99.1%, similar to those measured with SAR and PVdF.

**Figure 5:**
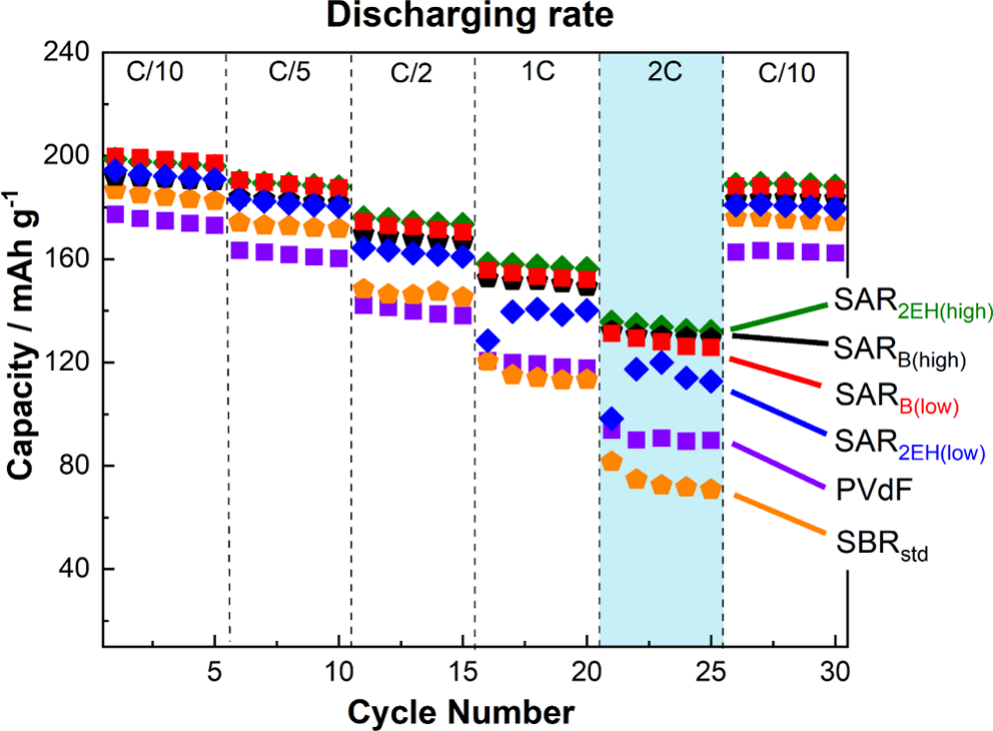
Rate capabilities of the LiNi_1/3_Mn_1/3_Co_1/3_O_2_//Li half-cells cycled at 25 °C with different binders in 1 mol dm^–3^ LiPF_6_ in EC/DMC. The respective rates increased from C/10 to 2C. The corresponding charge–discharge curves are shown in Figure S5.

Another important indicator for LIBs is rate capability. Figure 5 shows the rate capability of the electrodes fabricated with the different respective binders. The rate capability measurements were carried out at C/10, C/5, C/2, 1C, and 2C, and again at C/10. At the highest current density of 2C, the discharge capacities of the electrodes based on the SAR-based binders were higher than that of the PVdF-based electrode (90 mA h g^–1^). Because of the same conditions of particle morphology, crystallinity, and electrode loading of the NMC composite electrodes on the Al foil, we reasonably conclude that the improved affinity between the electrode and electrolyte promotes the movement of Li^+^ ions within the electrode and/or interface to increase the discharge capacity at high current densities.^28^

**Figure 6:**
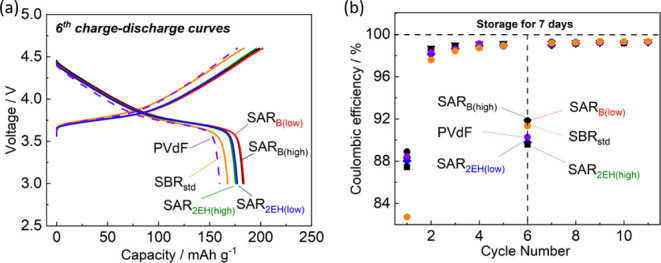
Self-discharge tests at 25 °C of the LiNi_1/3_Mn_1/3_Co_1/3_O_2_ electrodes fabricated with the different binders. (a) Charge–discharge curves for the 6th cycle and (b) Coulombic efficiency. The cells were cycled in 1 mol dm^–3^ LiPF_6_ in EC/DMC at 27.41 mA g^–1^ in the voltage range of 3.0–4.6 V and stored at 25 °C for 7 d after the 6th charge. The cycling was restarted after 7 d storage.

**Figure 7:**
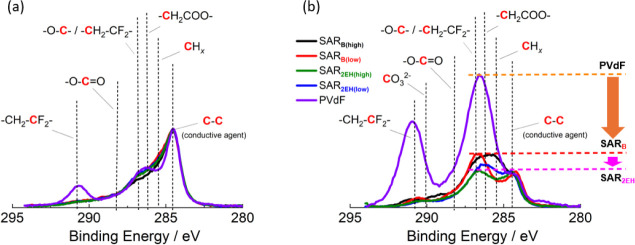
XPS C 1s spectra of the LiNi_1/3_Mn_1/3_Co_1/3_O_2_ composite electrodes with different binders (a) before and (b) after 50 cycles. The cells were cycled in the voltage range 3.0–4.6 V at 27.41 mA g^–1^ at 25 °C with 1 mol dm^–3^ LiPF_6_ in EC/DMC as the electrolyte.

The battery performance was further evaluated by conducting self-discharge tests of the fully charged electrodes with the different binders (Figure 6). The experimental conditions were as follows: after charging and discharging for up to 5 cycles, the electrode was fully charged for the sixth cycle and stored at the open circuit voltage at 25 °C for 7 d, after which the sixth discharge was restarted. As displayed in Figure 6, at the sixth cycle, the Coulombic efficiency of the electrodes with the SAR_B_-based binder was higher than that of the PVdF-based electrode; in particular, SAR_B_ had the highest value of >91.7%. As previously observed in self-discharge tests on SAR-based LiCoO_2_ electrodes, SAR_B(low)_ exhibited high Coulombic efficiency.^28^ This improvement is likely due to the effective passivation layer formed on the NMC particle surfaces with SAR, as explained in the subsequent discussion of the electrode-surface analysis results.^26^

The surface morphology of the composite electrodes prepared with different binders before and after cycling was compared through SEM observation. Figures S6 and S7 show the SEM images of the NMC111 electrodes with different binders before and after 50 cycles. Because these images did not reveal any significant differences among the binders, the pristine and cycled electrodes (50 cycles) were characterized through surface analysis via XPS. The cycled electrodes that were used for the measurements were washed with DMC and allowed to dry overnight in the glovebox.

Figure 7 shows the C 1s spectra of the electrodes with the different binders before and after 50 cycles. The pristine electrodes exhibited peaks derived from the different binders (e.g., −CF_2_– (291.1 eV) and −CH_2_–CF_2_– (286.6 eV)) and C–C (284.6 eV) derived from AB.^35,36^ In contrast, after 50 cycles, the intensities of the peaks corresponding to −O–C– (286.8 eV) and −CH_2_COO– (285.3 eV) for the electrode with PVdF were higher than that corresponding to C–C for this electrode. The marked intensification of these peaks is attributable to the formation of polycarbonate derivatives produced by the acidic decomposition of EC and DMC.^24,37−43^ A similar, yet much less pronounced, intensification was detected for the electrodes based on the SAR-based binders after cycling, in which the −O–C–, −CH_2_COO–, and −O–C=O– peak intensities also increased after cycling. The peak intensities of the electrolyte decomposition products decreased in the order of PVdF, SAR_B_, and SAR_2EH_. This suggested that the decomposition of the electrolyte solvent upon cycling is suppressed when the SAR-based binder is used.

**Figure 8:**
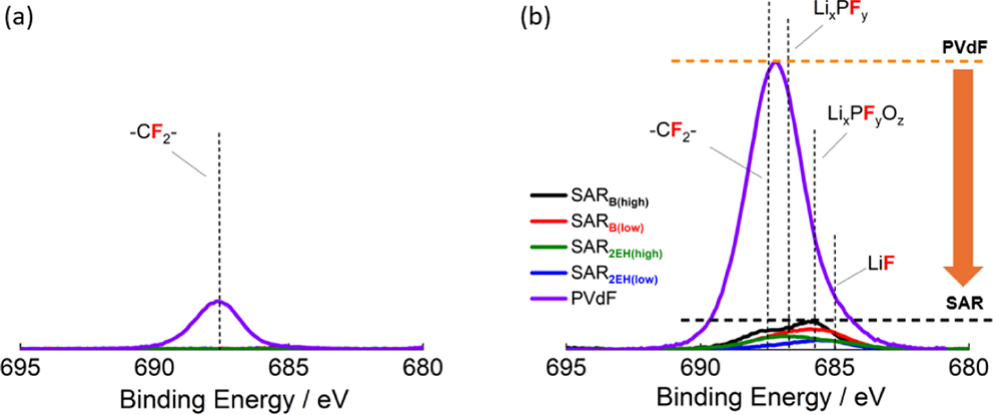
XPS F 1s spectra of the LiNi_1/3_Mn_1/3_Co_1/3_O_2_ composite electrodes with different binders (a) before and (b) after 50 cycles. The cells were cycled in the voltage range 3.0–4.6 V at 27.41 mA g^–1^ at 25 °C with 1 mol dm^–3^ LiPF_6_ in EC/DMC as the electrolyte.

Figure 8 shows the XPS F 1s spectra of the electrodes with different binders before and after 50 cycles. The peak of PVdF (−CF_2_−) or LiPF_6_ was observed at around ∼688 eV, whereas the peaks attributed to the decomposition products of LiPF_6_ (Li_*x*_PF_*y*_, Li_*x*_PF_*y*_O_*z*_, and LiF) were observed at ∼686.6 eV.^44−46^ For the pristine electrodes, only the peak ascribed to the PVdF binder was observed; however, after 50 cycles, the F 1s peak for the PVdF binder intensified drastically as a result of an increase in the amount of the electrolyte decomposition products. In contrast, the peaks corresponding to the electrolyte degradation products did not significantly intensify when SAR_B_ and SAR_2EH_ were used. These results suggest that the amount of the deposited electrolyte decomposition products increased with the number of cycles when PVdF was used. However, the formation of a stable passivation film during the initial cycle serves to suppress electrolyte decomposition for SAR-based electrodes.

Figure S8 presents the charge–discharge curves and cycling performance of the electrodes with PVdF, SAR_B(low)_, and SAR_2EH(low)_ during 100 cycles. The voltage drops of the electrodes with PVdF and SAR_2EH(low)_ is increased with cycling in comparison with that of the SAR_B(low)_-based electrode. We thought that this was attributed to the breakdown of the electrode structure with continuous cycling, partial isolation of active material particles, and a resultant increase in the internal resistance of the electrode due to the continuous electrolyte decomposition. Figure S9 shows the cross-sectional SEM images of the electrodes before and after cycling. After 100 cycles at 4.6 V, microcracks were observed on the SAR_2EH(low)_- and PVdF-based electrodes. In contrast, significantly fewer microcracks were observed on the SAR_B(low)_-based electrode. SAR_B(low)_ is considered to have protected the particle structure by effectively coating the particle surface with the deposition layer produced in the initial cycle. This is likely to have mitigated the internal strain and dissolution of transition metals during cycling to protect the particle structure.^20,47,48^

**Figure 9:**
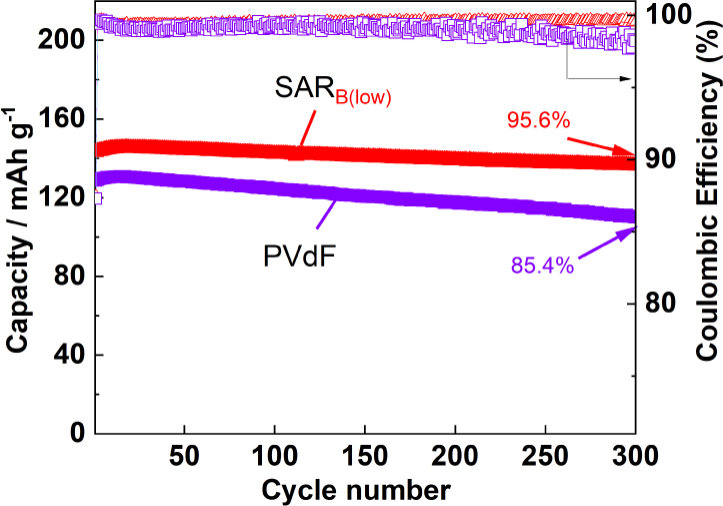
Long-term cycling performance and Coulombic efficiencies of the LiNi_1/3_Mn_1/3_Co_1/3_O_2_//Li half-cells with the different binders. The cells were cycled in the voltage range 3.0–4.2 V at 27.41 mA g^–1^ and 25 °C with 1 mol dm^–3^ LiPF_6_ in EC/DMC as the electrolyte. The corresponding charge–discharge curves are shown in Figure S10.

For the first 100 cycles, the SAR_B(low)_-based electrode exhibited the highest cycling performance among all the electrodes containing SAR as the binder. As shown in Figure S3, the capacity retention at 4.2 V was better than that at 4.6 V. Again, the upper cutoff voltage was set to 4.2 V to compare the long-term cycling stability of the electrodes with SAR_B(low)_ and PVdF. The results of the cycling tests for the NMC111 electrodes with the PVdF and SAR_B(low)_ binders (Figure 9) indicated that the SAR_B(low)_-based electrode outperformed the PVdF-based electrode in terms of both the capacity and Coulombic efficiency. Figure S10 shows the charge–discharge curves for the first, 100th, 200th, and 300th cycles. These curves show that the SAR_B(low)_-based electrode undergoes a lower polarization and suppresses discharge capacity degradation. In the initial cycle, the discharge capacity of the SAR_B(low)_-based electrode was higher than that of the PVdF-based electrode. As mentioned previously, this may indicate that the SBR_std_ and SAR-based binders form a stronger conductive network in the composite layer than that formed by PVdF.^34^ Furthermore, after 300 cycles, the SAR_B(low)_-based electrode exhibited a higher capacity retention rate of 95.6%. The decrease in the average Coulombic efficiency over 300 cycles in the order SAR_B(low)_ (99.53%) > PVdF (98.99%) suggests that SAR_B(low)_ facilitates the formation of a stable passivation layer in the initial cycle, which suppresses electrolyte decomposition, as discussed in Figure 8. This indicates that the formed passivation layer is effective in improving the reversibility as well as long-term stability, even for 300 cycles.

**Figure 10:**
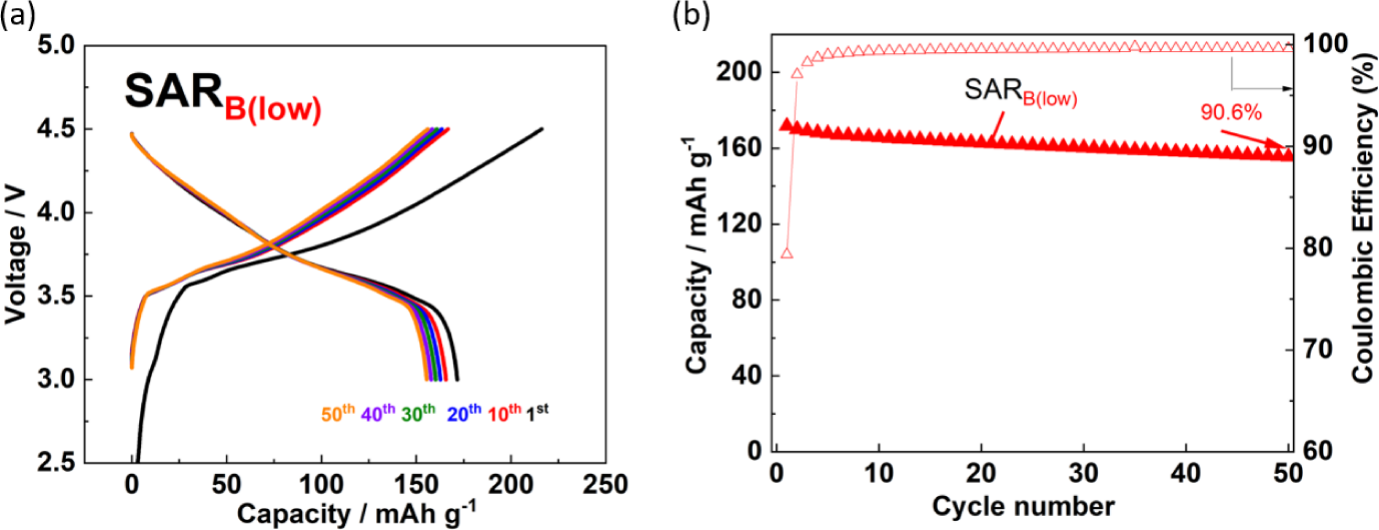
LiNi_1/3_Mn_1/3_Co_1/3_O_2_//graphite full-cells with SAR_B(low)_. (a) Charge–discharge curves. (b) Cycling performance and Coulombic efficiencies. The cells were cycled at 27.41 mA g^–1^ with 1 mol dm^–3^ LiPF_6_ in EC/DMC in the voltage range of 3.0–4.5 V.

The cycling performance of the NMC/graphite full-cells cycled at 4.3 V typically deteriorates, which has limited the use of commercial NMC as a high-voltage cathode material.^49,50^ This deterioration is due to the leaching of transition metal ions from NMC and their electrochemical reduction on graphite,^51^ thus promoting the growth of the SEI.^52−54^ The cycle performance of LiNi_1/3_Mn_1/3_Co_1/3_O_2_/graphite full cells with SAR-based binder was tested with setting the upper cutoff voltage of 4.5 V at which the electrodes are prone to degradation; results for the SAR_B(low)_ binder are shown in Figure 10. After 50 cycles, the cell retained 90.6% of its capacity. The amount of dissolved transition metals that were transferred to the negative electrode was assessed by conducting EDS of the graphite electrode surface after 50 charge–discharge cycles in the full cell. The atomic ratios of Mn, Co, and Ni on the graphite surface were 0.01%, 0.02%, and 0.00%, respectively (Table S1).^25^ This indicates that the deposition film that had formed during the initial cycles contributed to the suppression of transition metal dissolution.

**Figure 11:**
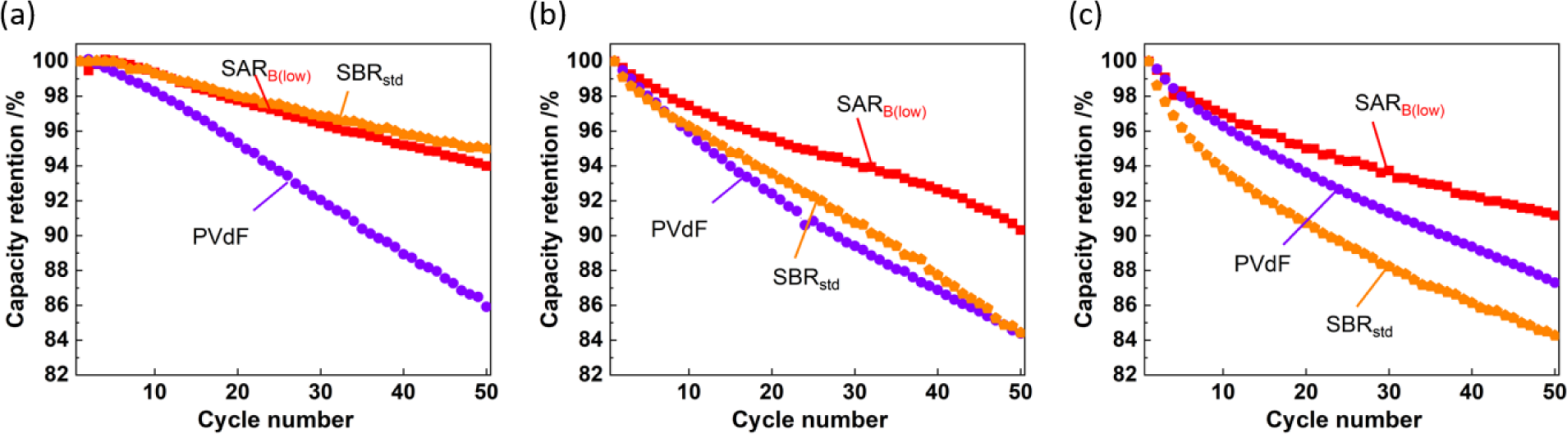
Capacity retention of the half-cells with the electrodes containing different binders and active materials with different Ni contents: (a) LiCoO_2_, (b) LiNi_1/3_Mn_1/3_Co_1/3_O_2_, and (c) LiNi_0.6_Mn_0.2_Co_0.2_O_2_ electrodes.

An increase in the nickel content of NMC is known to be accompanied by the reversible de/intercalation of a larger fraction of lithium ions within a constant voltage window.^55^ The performance of the high-Ni cathodes fabricated with the different binders was investigated by employing the LiNi_0.6_Mn_0.2_Co_0.2_O_2_ (NMC622) cathode material with 60% Ni content, which is a promising electrode material because its capacity is 12–16% higher than that of LiNi_1/3_Mn_1/3_Co_1/3_O_2_.^56,57^ In general, a high Ni content is typically associated with high reactivity between the active material and water, which degrades the cycling performance. We fabricated LiNi_0.6_Mn_0.2_Co_0.2_O_2_ electrodes with NMP-based PVdF and water-based SAR_B(low)_ and SBR_std_ binders and compared them to examine the effect of the binder on the cycling performance of the cells with these electrodes.^8−14^ The charge–discharge curves (Figure S11) confirmed that the SAR_B(low)_-based electrode is efficient in suppressing the degradation of the discharge capacity during the first 50 cycles. Figure 11 compares the capacity retention of the LiCoO_2_, LiNi_1/3_Mn_1/3_Co_1/3_O_2_, and LiNi_0.6_Mn_0.2_Co_0.2_O_2_ electrodes using different binders. The capacity retention of the PVdF-based electrodes with the aforementioned different active materials ranged from 84.4% to 87.3%; thus, the Ni content did not have a considerable influence on the cycling performance of the cells with these electrodes. For the SBR_std_ binder, the LiCoO_2_ electrode had the highest capacity retention, whereas that of the LiNi_*x*_Mn_*y*_Co_1–*x*–_*_y_*O_2_ electrode was significantly lower; in particular, the LiNi_0.6_Mn_0.2_Co_0.2_O_2_ electrode with SBR_std_ underwent severe degradation. Although the SBR_std_/CMC mixing ratio in the LiNi_*x*_Mn_*y*_Co_1–*x*–_*_y_*O_2_ and LiCoO_2_ electrodes was slightly different, this did not significantly affect the results, as shown in Figure S3. The C=C double bond in the structure is irreversibly oxidized during operation at high voltage, as shown in Figure 1. An increase in the Ni content of LiNi_*x*_Mn_*y*_Co_1–*x*–_*_y_*O_2_ concomitantly increases the particle volume change with cycling and promotes the surface catalytic oxidation of electrolytes and/or binders. Consequently, the protection of the particle surfaces is insufficiently maintained during cycling, resulting in the continuous decomposition of the electrolyte.^57^ In contrast, the capacity retention of the electrodes with the SAR_B(low)_ binder was >90% regardless of the Ni content of the active material. This is attributable to the excellent oxidative stability of the SAR-based binder and the particle surface coverage was similar to that of NMC111 as mentioned above.^28^

## Conclusion

SAR-based binders were synthesized and used to fabricate LiNi_1/3_Mn_1/3_Co_1/3_O_2_ electrodes for high-voltage operation. The use of the SAR_B_-based binders improved the mechanical properties of the corresponding electrodes. The XPS results revealed that the passivation film formed by each of the SAR-based electrodes in the initial cycle contributed to the inhibition of electrolyte decomposition. The extent to which the performance of the positive electrode incorporating active materials with different Ni contents (LiCoO_2_ and LiNi_0.6_Mn_0.2_Co_0.2_O_2_) is affected by the binder was studied. The results showed that SAR_B(low)_, which demonstrated the highest electrolyte uptake and flexibility, exhibited excellent cycling stability even with increasing nickel content.

## Supporting Information Available

Cyclic voltammograms, 90° peeling test, XPS profiles, effect of cycling on the upper voltage limit of LiNi_1/3_Mn_1/3_Co_1/3_O_2_//Li half-cells and different binder polymer contents, charge–discharge curves, cycling performances, SEM images, and surface atomic ratios estimated from the EDS profiles of graphite electrodes (PDF)

## Abbreviations

SAR: styrene-acrylic-rubber
DMC: dimethyl carbonate
EC: ethylene carbonate
NMC111: LiNi_1/3_Mn_1/3_Co_1/3_O_2_
NMC622: LiNi_0.6_Mn_0.2_Co_0.2_O_2_
XPS: X-ray photoelectron spectroscopy
LIB: lithium-ion battery
CMC: sodium carboxymethyl cellulose
NMP: *N*-methyl-2-pyrrolidone
SEM: scanning electron microscopy
EDS: energy dispersive X-ray spectrometry.
