# Supplementary material for: Electrochemical Properties of Powdery LiNi1/3Mn1/3Co1/3O2 Electrodes with Styrene-Acrylic-Rubber-Based Latex Binders at High Voltage
Source: ACS Appl Mater Interfaces. 2024 Nov 25;16(49):67577–86. doi: 10.1021/acsami.4c11185 (PMC11647888; doi:10.1021/acsami.4c11185)
Supplement: Supplementary file 1 — am4c11185_si_001.pdf [file am4c11185_si_001.pdf]

Supporting Information

Electrochemical Properties of Powdery $LiNi_{1/3}Mn_{1/3}Co_{1/3}O_2$ Electrodes with Styrene-Acrylic-Rubber-based Latex Binders at High Voltage

Lu Yin,[†] Ryoichi Tatara,[†,‡] Kosuke Nakamoto,[†] Shogo Yamazaki,[†] Rena Takaishi,[‡] Eisuke Shiiyama,[‡] Takashi Matsuyama,[‡] and Shinichi Komaba[†,*]

[†]*Department of Applied Chemistry, Tokyo University of Science, 1-3 Kagurazaka, Shinjuku, Tokyo 162-8601, Japan*

[‡]*NIPPON A&L INC., 3-1-98 Kasugadenaka, Konohanaku, Osaka, 554-8558, Japan*

[*]*Correspondence to: komaba@rs.tus.ac.jp*

[‡]*Current address: Department of Chemistry & Life Science, Yokohama National University, 79-5 Tokiwadai, Hodogayaku, Yokohama 240-8501, Japan*

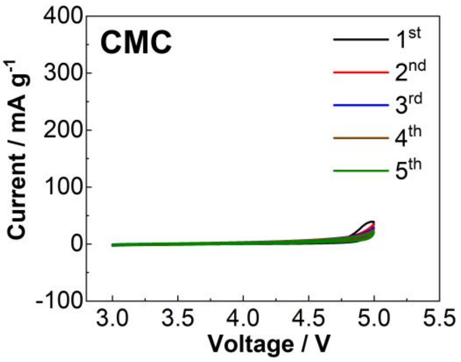

Figure S1. Cyclic voltammograms of the $LiNi_{1/3}Mn_{1/3}Co_{1/3}O_2$-free composite electrode containing 80 % acetylene black (AB) and 20 % CMC with 1 mol dm^{-3} $LiPF_6$ in EC/DMC as the electrolyte in the voltage range of 3.0–5.0 V measured at 25 °C.

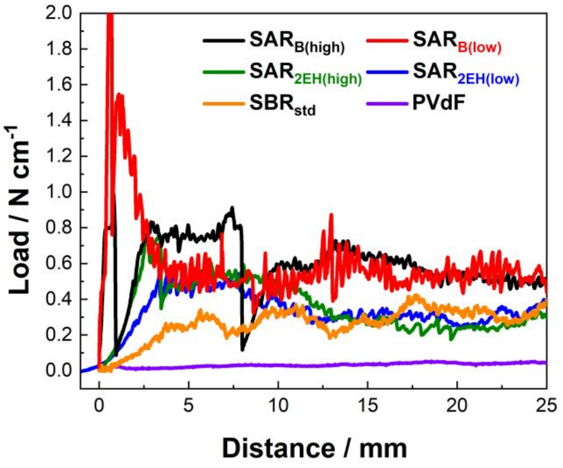

Figure S2. 90° peeling test results for the $LiNi_{1/3}Mn_{1/3}Co_{1/3}O_2$ electrodes. The average load was calculated from the stable plateau regions, typically after ~10 mm.

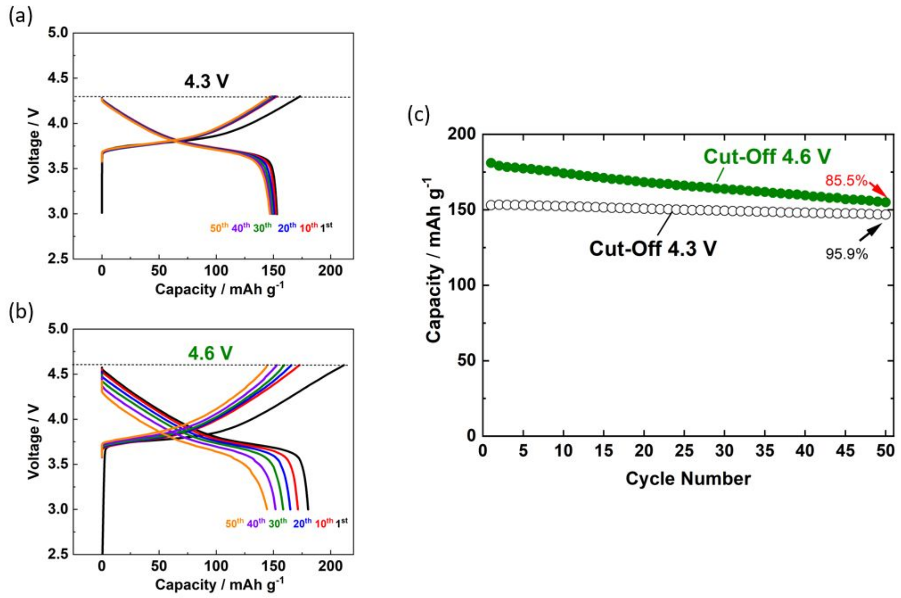

Figure S3. Performance of the $LiNi_{1/3}Mn_{1/3}Co_{1/3}O_2$ composite electrodes with PVdF binder; (a) and (b) charge–discharge curves and (c) reversible capacity in the voltage range of 3.0–4.6 V at a rate of 27.41 mA g^{-1}.

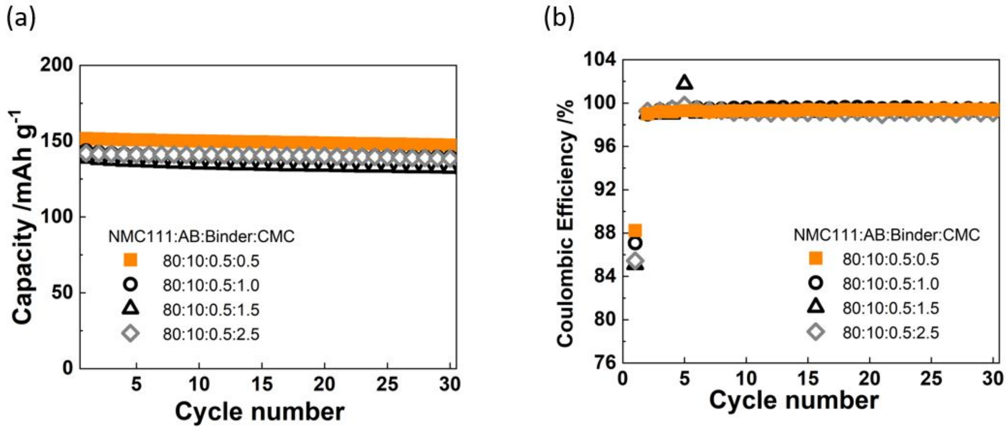

Figure S4. Variations in the (a) capacities and (b) Coulombic efficiencies of the $LiNi_{1/3}Mn_{1/3}Co_{1/3}O_2$//Li half-cells with SBR_{std} at different contents of binder polymer in the composite electrodes. The cells were cycled in the voltage range of 3.0–4.6 V at 25 °C with 1 M $LiPF_6$ in EC/DMC as the electrolyte.

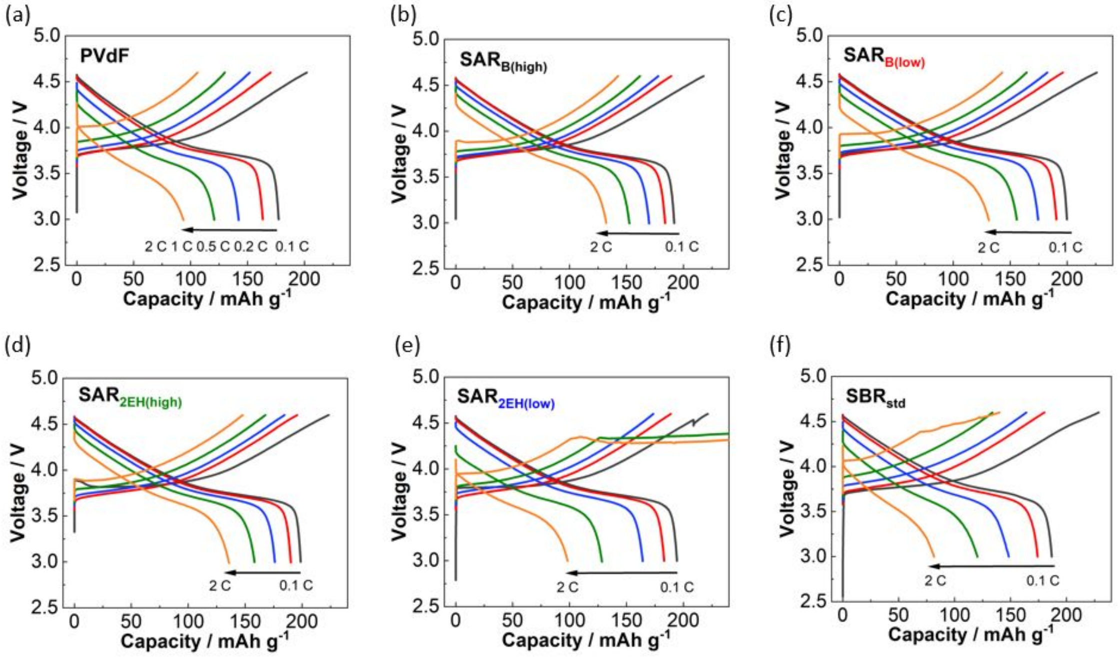

Figure S5. Charge and discharge curves of the $LiNi_{1/3}Mn_{1/3}Co_{1/3}O_2$//Li half-cells with the (a) PVdF, (b) $SAR_{B(high)}$, (c) $SAR_{B(low)}$, (d) $SAR_{2EH(high)}$, (e) $SAR_{2EH(low)}$, and (f) SBR_{std} binders. The cells were cycled at 25 °C with 1 mol dm^{-3} $LiPF_6$ in EC/DMC as the electrolyte.

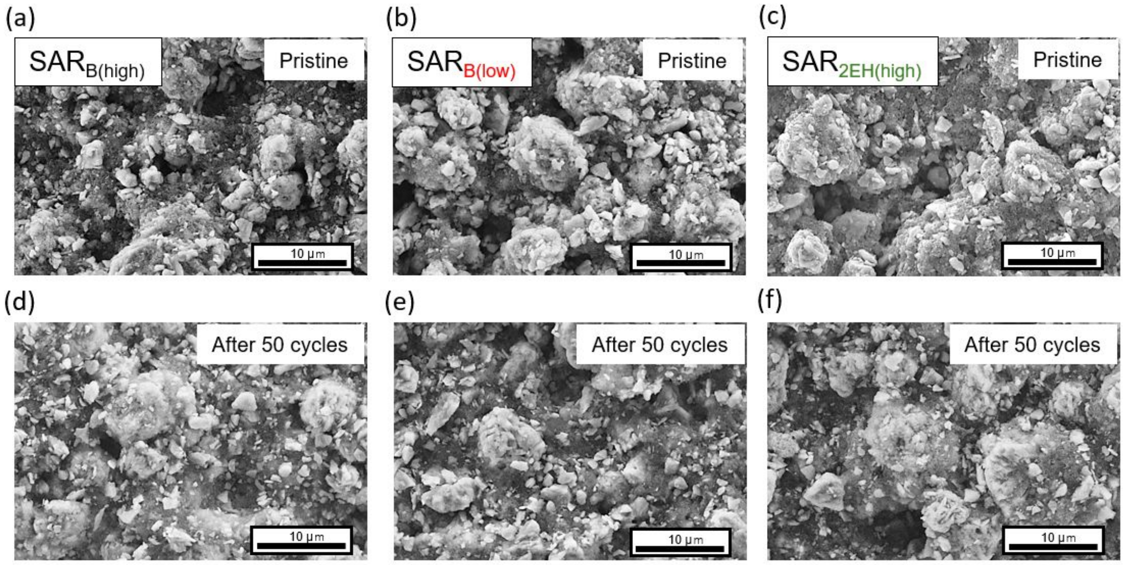

Figure S6. SEM images of the $LiNi_{1/3}Mn_{1/3}Co_{1/3}O_2$ electrodes with the (a,d) $SAR_{B(high)}$, (b,e) $SAR_{B(low)}$, and (c,f) $SAR_{2EH(high)}$ binders before and after 50 cycles at 25 °C.

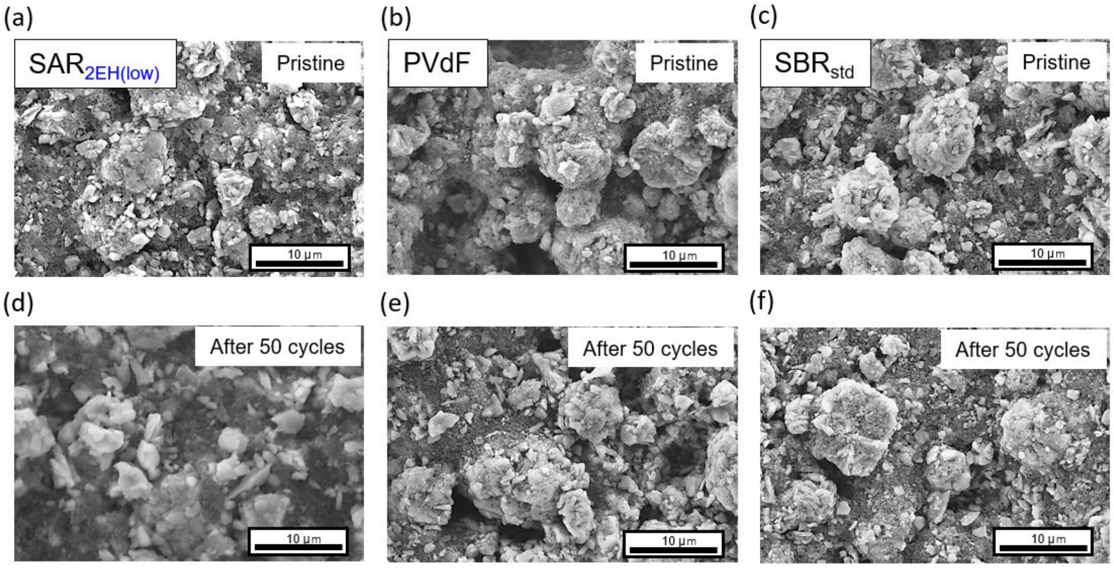

Figure S7. SEM images of the $LiNi_{1/3}Mn_{1/3}Co_{1/3}O_2$ electrodes with the (a,d) $SAR_{2EH(low)}$, (b,e) PVdF, and (c,f) SBR_{std} binders before and after 50 cycles at 25 °C.

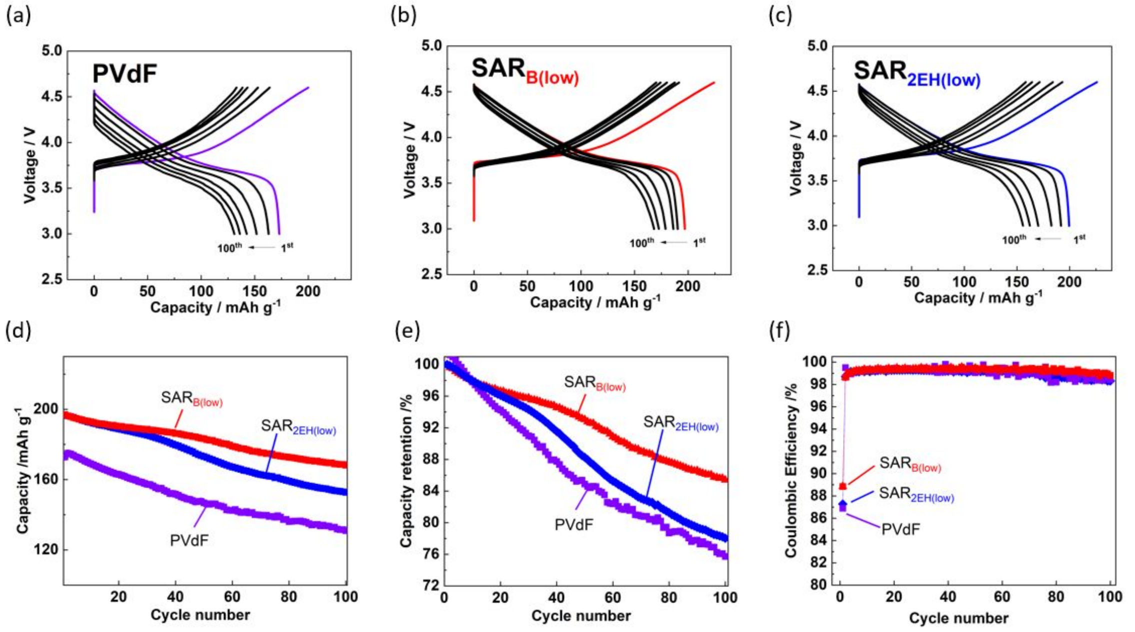

Figure S8. Charge–discharge curves of the $LiNi_{1/3}Mn_{1/3}Co_{1/3}O_2$//Li half-cells with the (a) PVdF, (b) $SAR_{B(low)}$, and (c) $SAR_{2EH(low)}$ binders. (d) Discharge capacities, (e) capacity retention, and (f) Coulombic efficiencies. The cells were cycled (100 cycles) at 27.41 mA g^{-1} at 25 °C with 1 mol dm^{-3} $LiPF_6$ in EC/DMC as the electrolyte.

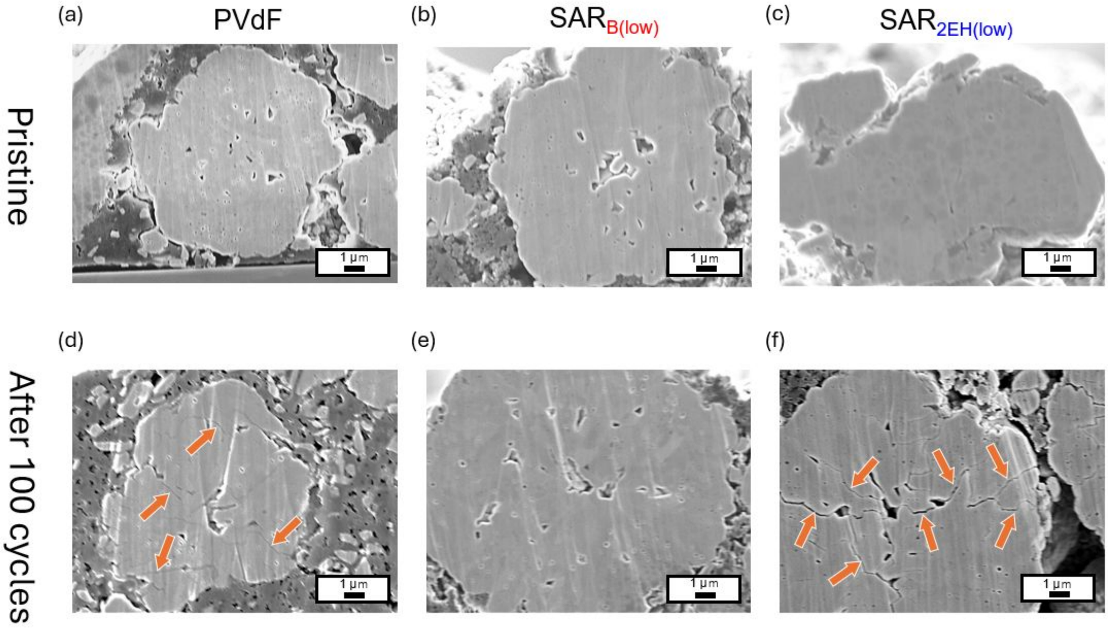

Figure S9. Cross-sectional SEM images of the $LiNi_{1/3}Mn_{1/3}Co_{1/3}O_2$ electrodes with the (a,d) PVdF, (b,e) SAR$_{B(low)}$, and (c,f) SAR$_{2EH(low)}$ binders before and after 100 cycles at 25 °C.

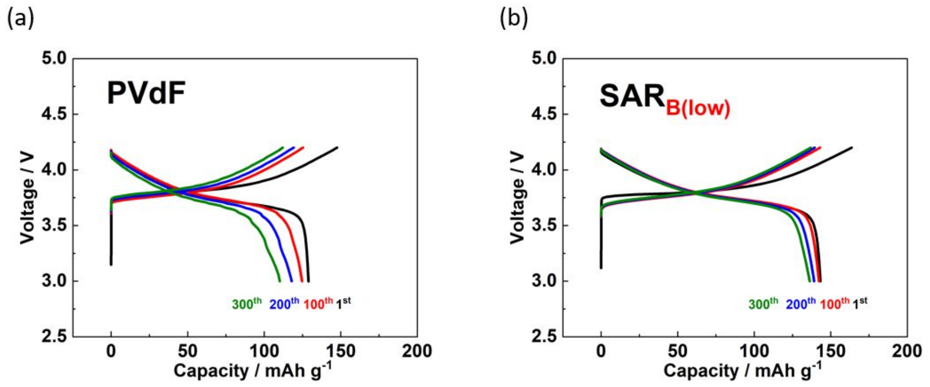

Figure S10. Charge–discharge curves of the $LiNi_{1/3}Mn_{1/3}Co_{1/3}O_2$//Li half-cells with the (a) PVdF and (b) $SAR_{B(low)}$ binders. The cells were cycled at 27.41 mA g^{-1} at 25 °C with 1 mol dm^{-3} $LiPF_6$ in EC/DMC as the electrolyte.

Table S1 Surface atomic ratios estimated from the EDS results of the graphite composite electrode after 50 cycles in the $LiNi_{1/3}Mn_{1/3}Co_{1/3}O_2$//graphite full-cell at 25 °C with $SAR_{B(low)}$.

Element	Atomic ratio / %
C	97.54
O	0.47
F	0.58
P	1.08
Mn	0.01
Co	0.02
Ni	0.00
Cu	0.31
Total	100

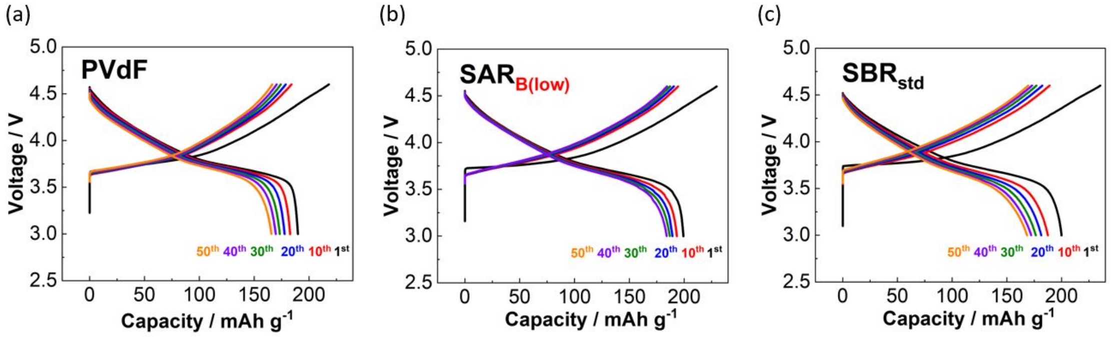

Figure S11. Charge–discharge curves of the $LiNi_{0.6}Mn_{0.2}Co_{0.2}O_2$//Li half-cells with the (a) PVdF, (b) $SAR_{B(low)}$, and (c) SBR_{std} binders. The cells were cycled at 27.6 mA g^{-1} with 1 mol dm^{-3} $LiPF_6$ in EC/DMC in the voltage range of 3.0–4.6 V.
